# Reduced Sensitivity of Older Adults to Affective Mismatches

**DOI:** 10.1100/tsw.2007.115

**Published:** 2007-05-29

**Authors:** Yang Jiang, Victoria Vagnini, Jessica Clark, Qin Zhang

**Affiliations:** ^1^ Department of Behavioral Science, University of Kentucky, Lexington, USA; ^2^ Department of Psychology, University of Kentucky, Lexington, USA; ^3^ Department of Psychology, The Capital Normal University, Beijing, China

**Keywords:** emotion, cognition, positive valence, neutral valence, negative valence, evaluative task, pleasantness, semantic, IAPS, ANEW, implicit learning, prime-target, reaction time, brain aging, mood, congruency, conflicts

## Abstract

The present study investigated age-related differences in emotional processing by using a paradigm of affective priming. Eighteen, right-handed, younger (mean age 22) and 15 older (mean age 68) subjects pressed buttons to indicate pleasantness of target words. The valence of each prime-target pair was congruent (e.g., win-love), incongruent (e.g., love-loss), or neutral (time-flower). Two sets of 720 prime-target pairs used either affective words or pictures as primes, and affect words as targets. We included well-matched positive and negative valence pairs in all congruent, neutral, and incongruent conditions, and controlled for possible contamination by semantic meaning, word frequency, and repetition effects. The response time (RT) results revealed that young participants responded faster to the targets in affectively congruent conditions than in incongruent conditions. In older participants, the responses to target words were indifferent to all valence congruency conditions. The age effect in affective priming largely reflects reduced sensitivity to affective mismatches among older adults. Intriguingly, emotional Stroop effect and some perceptual priming have been linked to increased interferences and mismatches in older adults. The age-related changes in affective, perceptual, and semantic processes are discussed.

## INTRODUCTION

Cognitive impairment in older adults and dementia patients is often accompanied by changes in mood and affective processing. Recent evidence from behavioral and neuroimaging studies have shown a mixed picture of age-related changes in affective processing on cognitive processes, such as attention, memory, and judgments[1,2,3,4].

One approach to studying emotional processes is to use an affective priming paradigm. Priming refers to the phenomenon where previous experience with a stimulus (e.g., word or picture) biases or alters subsequent behavior, producing, for example, faster reaction times, improved accuracy, or biased responses to the same or a similar stimulus. Priming is often regarded as a particular kind of implicit memory and learning[5]. For instance, processing of a positive target word (e.g., love) is faster and more accurate when it is preceded by a positive word (e.g., win) rather than a negative word (e.g., lose). Specifically, the time needed to evaluate a target word as either affectively "positive" or "negative" is shorter when prime and target words are affectively congruent (positive-positive or negative-negative) than when they are affectively incongruent (positive-negative). This effect is referred to as *affective priming*[6,7,8]. Recently, researchers in psychology and clinical neuroscience have applied tasks of affective priming (prime-target) as tools for studying emotional processing of normal, healthy, young adults[6,9,10,11,12,13], schizophrenia patients[14], and patients with neuroticism[15].

Other types of priming, such as semantic priming, repetition priming, and perceptual priming, have been examined in both young and older adults. In contrast to recognition memory, semantic priming (facilitation in semantic-related word pairs, e.g., student-teacher) has been shown to vary little with age[16]. Whether, and to what extent, repetition priming and perceptual priming are age sensitive remain controversial[17,18,19]. So far, there have been limited studies on aging and visual affective priming[20,21,22]. Some aging and affective studies have shown that the elderly have more difficulty than the young in overcoming the influence of the negative affect, yet other studies suggest that older adults focus more on positive affects[1,3,4,21,23]. Instead of focusing on positive priming and negative priming, the present study examined whether there is an age effect in processing affective congruency and mismatches, including both positive and negative valence words in primes and targets.

Various behavioral tasks have been applied to examine implicit affective processing, including evaluative categorization tasks[9,10,11], naming tasks[6,7,12,24,25], and lexical-decision tasks[9,26,27,28]. Since a previous semantic categorization task failed to find an affective priming effect[29], the present study chose an evaluative categorization task to examine age effects on performance of affect priming. In the current study, reaction times were calculated while the young and older participants pressed buttons to indicate whether the target word was pleasant or unpleasant. We carefully controlled the prime-target pairs for valence dimension and matched semantic meaning, word frequency, and arousal dimensions of the primes and targets. In addition, both pictures and words were included as primes to ensure that any age effect on affective priming is not due to the choice of visual stimuli.

## METHODS

### Participants

Eighteen, right-handed, younger (M = 22.39, SD = 3.33, range = 18–28) and 15 older (M = 68.20, SD = 3.14, range = 64–75) subjects were recruited from the Lexington, Kentucky area. Inclusion criteria consisted of normal or corrected-to-normal vision, a score of 10 or below on the Beck Depression Inventory, and English as the native language.

### Materials

Two sets of 720 prime-target pairs (240 affectively congruent, 240 incongruent, and 240 neutral) were selected. Each target word was primed by either a word or picture (Fig. 1). There was no semantic or associative relationship between the prime and target, apart from affect. All the target words were selected from Affective Norms for English Words (ANEW; [43]). Using a Likert scale (1-9 with 9 being the most positive), there was a significant difference between positive (M = 7.64) and negative (M = 2.44) affective words. The word primes consisted of 120 positive (M = 7.61), 120 negative (M = 2.38), and 240 neutral (M = 5.09; ANEW; [43]) words. Similarly, picture primes were 120 positive (M = 7.49), 120 negative (M = 2.52), and 80 neutral (M = 4.98) pictures selected from the International Affective Picture System (IAPS; [44]). All target words were used six times throughout the paradigm, each positive or negative prime was used twice, each neutral prime picture was used three times, and each neutral prime

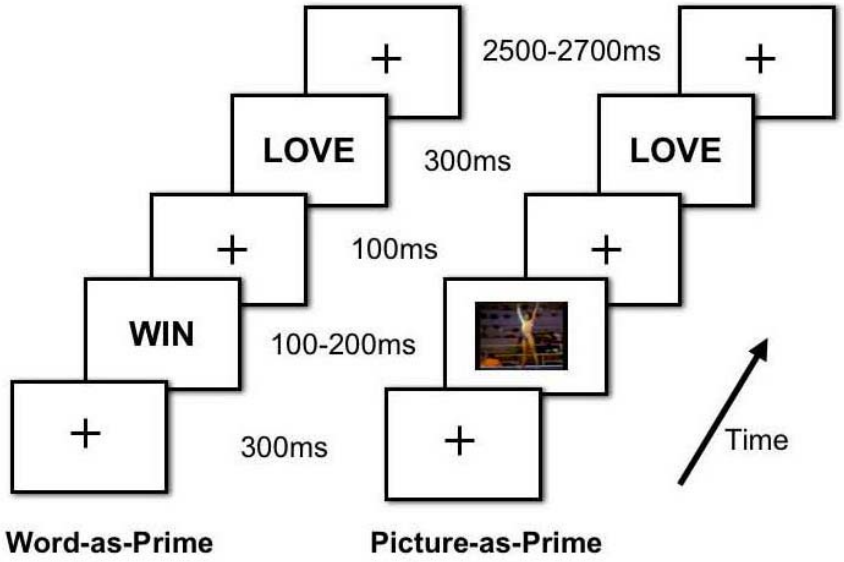

**FIGURE 1.** The affective priming task and timing of stimuli presentation. The left panel shows a trial using a word as a prime. The right panel illustrates a trial using a picture as a prime.

word was used once. In order to reduce the repetition times of primes and targets, each participant received one-third of each type of prime-target pairing. Therefore, each participant only had targets repeatedly presented twice (i.e., targets in the picture-as-prime condition were the same as targets in the word-as-prime condition).

As shown in Fig. 1, each trial began with a 300 ms fixation cross, followed by a priming stimulus (100 ms for words, 200 ms for pictures). This was then replaced by the fixation cross for 100 ms, then a target word was presented for 300 ms followed by a 2500–2700 ms presentation of another fixation. The presentation order of picture-prime and word-prime trials was counterbalanced.

## Task

Each participant was told to observe the first item on the screen (prime) and only respond to the second item (target) by indicating whether it was pleasant or unpleasant. Participants pressed a “happy” or “sad” button to indicate their preference. They were told to make their judgment as quickly and as accurately as possible. A 2-min break was provided after each 60-trial block in order to offset excessive eye movement. Each participant underwent a 60-trial practice session before the testing session.

## Data Analysis

A four-way repeated measures analysis of variance (ANOVA), 2 age (younger or older) × 2 prime stimuli (pictures or words) × 2 target valence (positive or negative) × 3 affective congruency (congruent, incongruent, or neutral), was conducted. Bonferroni correction was applied for multiple comparisons, and all $p$ values were corrected using the Greenhouse-Geisser correction when appropriate. Trials that were inconsistent with the previously determined norm, or had RT values outside a 350–2000 ms range, were excluded from mean RT calculations (about 2% of trials).

## RESULTS

There were no significant differences in demographic variables, such as gender, race, or education, between the young and older groups ($p > 0.05$). A difference was found on marital status, i.e., none of the younger group was married, while all participants in the older group had been married at least once in their lifetime, with many currently divorced or widowed. A difference was also found in socioeconomic status, with younger individuals more likely to report that they were able to afford “necessities and low cost extras”, while the older adults were more likely to be able to afford “expensive extras”.

Scores on the Beck Depression Inventory showed no significant group differences between older (M = 4.47, SD = 3.85) and younger subjects (M = 3.50, SD = 3.20). These values are not indicative of clinical problems with depression.

Significant main effects were found for age, valence, and congruency conditions ($p < 0.01$). Older subjects had slower reaction times (M = 843 ms, SD = 150 ms, SE = 32 ms) than the younger subjects overall ($d$ = 5.56, M = 672 ms, SD = 117 ms, SE = 29 ms), $F$ (1,31) = 15.281, $p < 0.001$. Whether the prime was a picture or word did not produce any significant difference in reaction times to target words ($p > 0.10$; Fig.2). This result suggests that the valence, rather than the format of the stimulus, primes the target affect.

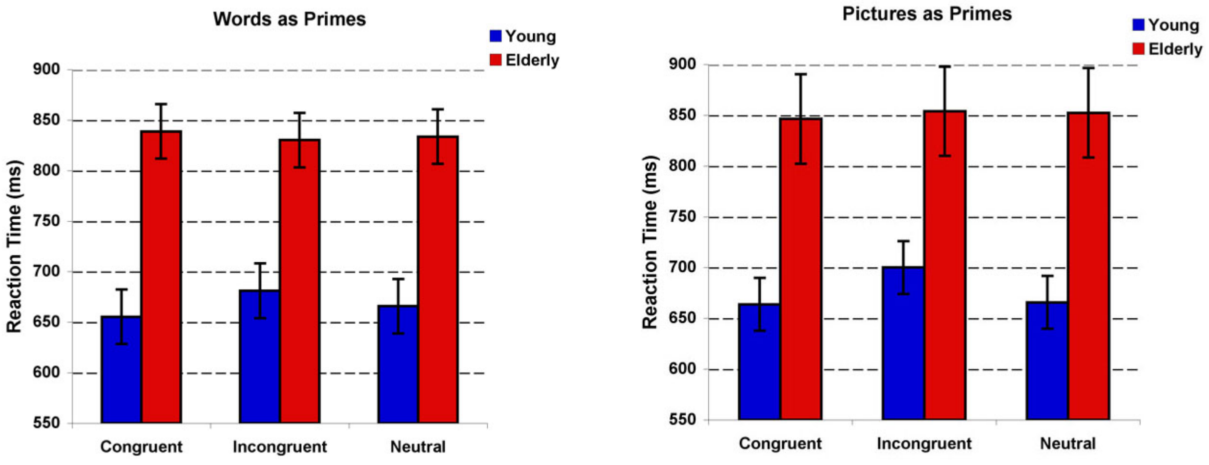

**FIGURE 2.** A. Comparison of mean reaction times (milliseconds) for older and younger subjects, with words used as primes (left) or with pictures as primes (right), as a function of affective condition (congruent, incongruent, and neutral). Error bars indicate ±1 standard error.

The main focus of the current investigation is age effect on affective congruency. Importantly, there was a significant age × affective congruency interaction, $F$ (2,30) = 8.844, $p < 0.01$, in either primed condition Analysis of simple effects indicated that younger participants responded more slowly to affectively incongruent pairs (M = 690.5 ms, SD = 126 ms, SE = 31 ms) than they did to congruent or neutral conditions (M = 659.5 ms, SD = 114 ms, SE = 29, $d$ = 1.03; and M = 666, SD = 113 ms, SE = 29 ms, $d$ = .82 respectively). However, older adults showed no significant difference among the all congruency conditions (Fig. 3).

Fig. 3 shows the age effect on affect priming, commonly defined as comparison between congruent and incongruent conditions. The comparisons between congruent vs. neutral condition and incongruent vs. neutral condition in both young and older adults are also shown. Post hoc analyses indicated no significant age-related differences for the congruent vs. neutral condition; however, a significant age effect was found for incongruent vs. neutral, in which younger participants were sensitive to affective mismatches while older adults were not. The reduced affective priming seen in Fig. 3 is, therefore, mostly contributed by reduced sensitivity to affective mismatches in the older adults.

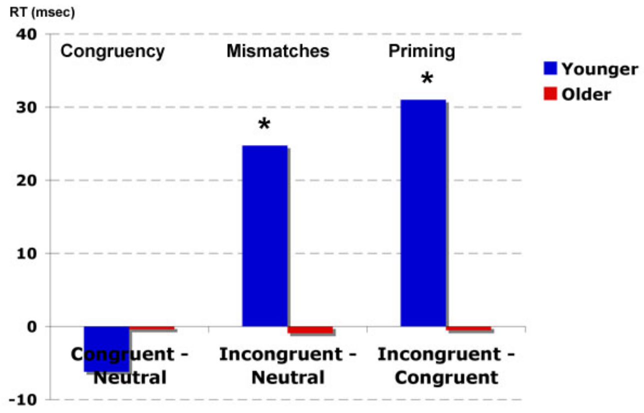

**FIGURE 3.** Age and the RT difference between conditions. Right: index of affect priming; RT difference between congruent and incongruent conditions for young and older adults. Center: affective mismatch, i.e., RT difference between incongruent and neutral conditions for young and older adults. Left: affective congruency, i.e., significant RT difference between congruent and neutral conditions for young and older adults. Note the dominant contribution of a strong affective mismatch effect to the substantial affect priming effect in the younger subjects. By contrast, dependence of reaction times on congruency conditions is negligible.

## DISCUSSION

The results indicate substantial age-related differences in processing affective stimuli. Older adults did not show differences in reaction times among congruent, incongruent, and neutral affective conditions, an indication of absence of affective priming. When the valence of the prime and target differed, responses of younger subjects to targets were delayed. Sensitivity to affective conflicts or mismatches is not preserved in the older participants. Though not significant, the young also appeared to show a slightly faster response to affective congruent pairs compared to the neutral condition.

One may speculate that the reduced influence of affective mismatches among the elderly was due to the simple fact that any prime-target interferences are reduced in older adults. If so, the current results have nothing to do with affective mismatches *per se*. Interestingly, previous studies on aging and conflict effects, such as Stroop interfering studies, have shown that interference is actually larger for older than for younger adults (e.g., [30]). Young subjects did not benefit from congruence nor suffer from incongruence[31]. However, the age effects on affective priming found in the present study showed the opposite trend, i.e., the young individuals do suffer from affective incongruence.

Note that age and positive or negative valence effects should be interpreted with caution. Affective priming effects can be reversed by many factors, such as word frequency or familiarity[13]. Our study included both positive and negative valence pairs in all congruent, neutral, and incongruent conditions. Possible contamination of semantic priming, word frequency, and repetition effects were also controlled. The present results provide evidence that older adults lose sensitivity to mismatches of affective stimuli, regardless of preferences in positive or negative valence.

Is it possible that the age effect on affective priming is really caused by response bias or time-related priming? Unlike semantic priming or visual repetition priming that can last for minutes or days[17,32,33], affective priming effects are short lived. An affective prime and a target must be presented closely in time (typically within 300 ms) to achieve this effect[8,10,25,34]. A recent study by Ready et al.[22] compared affective priming and response timing, controlling for individual differences in speed, speed variability, and motor priming. They reported that older adults exhibited stronger valence-related priming effects and weaker time frame–related priming effects, relative to younger adults.

Furthermore, age-related differences in short-lived perceptual priming have been reported in visual motion priming studies. In contrast to affective priming, these studies reported increased perceptual mismatches in elderly subjects during perception of motion directions and rotations, over similar time frames of motion priming tasks[35,36]. This suggests that perceptual priming cannot account for the age dependence of affective priming. It is also known that older individuals exhibit decreased cognitive and executive control[37]. Though the current affective priming task is an implicit task, we cannot rule out the possibility that the age effect for affective mismatches is related to reduced inhibition in the elderly.

Another possibility is that stimuli presentation was not long enough for the elderly to process the valence of primes or targets fully. In this case, all the affective conditions might be processed indifferently in older adults. In other words, the stimulus onset asynchrony (SOA, the interval between the onset of the prime and the onset of the target) of 200–300 ms might be just enough to create affective priming effect only in young observers, whereas they were too brief for the elderly to access the emotional content of words or pictures. A significant interaction between SOA and target valence in older adults has been reported in a recent study by Leclerc and Hess[21]. They found that negative targets were judged significantly faster than positive targets at SOAs of 250 and 450 ms, but not at 900 ms. They inferred that the privileged status of negative information is most evident at earlier points of attention and visual information processing. They also suggested that older adults might be able to overcome initial interference from negative information when more time to process the prime words is permitted. Future studies should test the effect of SOA on affective priming in older adults by extending the presentation time of primes and targets.

Neural mechanisms underlying various priming tasks may reflect distinct processes in the brain. The faster reaction time associated with repetition priming has been linked to reduced neural activity in the brain, measured with functional MRI[38,39,40,41]. One recent investigation used event-related potentials (ERPs) to investigate the underlying neural mechanisms of visual affective priming in young participants[42]. Compared to neutral trials, affectively incongruent trials had larger and more N200 activation. In addition, a delayed N400 for word prime-target pairs matched the RT results, with larger amplitudes for incongruent than congruent pairs. This finding suggests that the affective priming occurs as early as 200–400 ms in the brain. These results lead to the prediction that older adults should evoke reduced ERP responses to affective mismatches.

To sum up, the present study investigated the age-related difference in processing of affective stimuli. We found that sensitivity to affective mismatches was reduced in the elderly when primes and targets were closely presented, within 300 ms. Understanding age-related changes in emotional processes is the first step to identifying indicators of cognitive and emotional changes that are linked to old-age dementias. Neuroimaging studies on aging and affect should provide insights on when and where such changes occur in the brain. Future studies should investigate the neural basis of reduced affective priming in the aged brain.

## ACKNOWLEDGMENTS

Part of the results was presented at the 2005 Annual Meeting of American Psychological Association. This project was supported by NIH K01AG00986, and part of NIH P50AG05144-21 pilot grant to YJ. We thank A. Morgan for assistance in data collection, M. Posner and an anonymous reviewer for helpful comments.
